# The role of cine magnetic resonance imaging in the evaluation of uterine contractility in patients with deep infiltrating endometriosis

**DOI:** 10.1590/0100-3984.2023.56.3e1-en

**Published:** 2023

**Authors:** Luís Ronan Marquez Ferreira de Souza, Patricia Prando Cardia

**Affiliations:** 1 Associate Professor of Radiology at the Universidade Federal do Triângulo Mineiro, Coordinator of the Female Pelvic Imaging Sector of the Colégio Brasileiro de Radiologisa e Diagnóstico por Imagem. Email: luisronan@gmail.com.; 2 Radiologist and Coordinator of the Program for Medical Residency and Improvement in Radiology at the Hospital Vera Cruz and the Hospital da Pontifícia Universidade Católica de Campinas. Email: patriciaprando@gmail.com.

Imaging evaluation is a frequent and essential practice in the clinical management of female infertility and for the guidance of infertile couples. The investigation of the most common causes of female infertility includes the characterization of congenital anomalies of uterine morphology, by transvaginal ultrasound and magnetic resonance imaging (MRI); the evaluation of tubal patency by hysterosalpingography and magnetic resonance hysterosalpingography; and the evaluation of ovarian reserve and other clinical conditions, such as endometriosis, adenomyosis, and uterine fibroids. In recent years, there have been a number of studies employing cine MRI for the evaluation of uterine contractility, demonstrating altered uterine peristalsis in patients with endometriosis, adenomyosis, or uterine fibroids^(1,2)^.

The article “Deep infiltrating endometriosis: cine magnetic resonance imaging in the evaluation of uterine contractility”, authored by Soares et al.^(3)^ and published in this issue of Radiologia Brasileira, describes the use of cine MRI in a 3.0-T scanner, for the evaluation of uterine peristalsis by characterizing the contractile movements of the uterus in patients with uterine endometriosis and adenomyosis. The authors evaluated 43 patients, of whom 18 had a radiological diagnosis of endometriosis. They found that, in the periovulatory and luteal phases, uterine peristalsis was more common among patients with deep endometriosis than among those in the control group. Among the patients diagnosed with adenomyosis, uterine contractility was significantly lower that that observed for the patients in the control group.

Soares et al.^(3)^ carried out a prospective study and expanded the exclusion criteria, which is one of the strengths of their article, with attention to factors that are known to affect uterine peristalsis, such as the use of hormonal contraceptives, the use of intrauterine devices, having amenorrhea ^(4)^, and being in the menstrual phase. Excluding patients who presented with any of those factors increased the robustness of the findings. It is questionable whether the use of those exclusion criteria was responsible for the fact that Soares et al.^(3)^ obtained results that diverge from those of previous studies on the topic, in which uterine peristalsis was reported to be less common in patients with endometriosis^(5)^. The authors’ choice not to use antispasmodics (which could affect uterine contractility) prior to the MRI examination is evidence of the care taken in order to obtain reliable results, as well as demonstrating the need to adapt the timing of the administration of that medication, which is routinely used during female pelvic examinations at most imaging centers, in order to minimize artifacts related to intestinal peristalsis.

The main limitations of the Soares et al.^(3)^ study, as noted by the authors themselves, are the relatively small size of the sample evaluated and the fact that the statistical analysis did not reveal a significant result regarding changes in peristalsis in patients with endometriosis. Their research could be expanded upon in studies involving larger patient samples and correlation with the different phases of the menstrual cycle, as well as evaluation of the impact of contractility.

Soares et al.^(3)^ demonstrated that the functional evaluation of uterine contractility by MRI is emerging as a promising field of research, which will undoubtedly enhance the investigation of female infertility^(6,7)^. It is also possible that this approach will improve the quality of cine MRI sequences acquired in 1.5-T scanners, thus making such evaluations accessible to more patients.

## References

[1] Kido A, Togashi K (2016). Uterine anatomy and function on cine magnetic resonance
imaging. Reprod Med Biol.

[2] Soares DM, Werner Junior H, Bittencourt LK (2019). The role of cine MR in the assessment of uterine
function. Arch Gynecol Obstet.

[3] Soares DM, Bittencourt LK, Lopes FPPL (2023). Deep infiltrating endometriosis: cine magnetic resonance imaging
in the evaluation of uterine contractility. Radiol Bras.

[4] Fornazari VAV, Salazar GMM, Vayego SA (2019). Impact of uterine contractility on quality of life of women
undergoing uterine fibroid embolization. CVIR Endovasc.

[5] Kido A, Togashi K, Nishino M (2007). Cine MR imaging of uterine peristalsis in patients with
endometriosis. Eur Radiol.

[6] Bazot M, Daraï E (2017). Diagnosis of deep endometriosis: clinical examination,
ultrasonography, magnetic resonance imaging, and other
techniques. Fertil Steril.

[7] Leyendecker G, Kunz G, Wildt L (1996). Uterine hyperperistalsis and dysperistalsis as dysfunctions of
the mechanism of rapid sperm transport in patients with endometriosis and
infertility. Hum Reprod.

